# P83 - Asthma inflammatory subtype specific treatment; a randomised clinical study

**DOI:** 10.1186/2045-7022-4-S1-P138

**Published:** 2014-02-28

**Authors:** Doaa Youssef, Rabab Elbhedy, Hosam Salah

**Affiliations:** 1 Zagazig University, Zagazig, Egypt

## Introduction

Macrolides antibiotics, such as clarithromycine express immunomodulatory and tissue reparative effects that are distinct from their anti-infective properties, and have in vitro efficacy against neutrophils.

## Aim of study

To determine the efficacy of add-on therapies that target eosinophilic and noneosinophilic airway inflammation and their effects on asthma control test, pulmonary function and asthma symptoms.

## Methods

single blind randomized clinical trial; asthmatic children with persistent symptoms undergoing treatment with fluticasone 100 mg bid and β2 agonist as required were studied. Group A (23 males / 17 females, aged 11.5±1.8 years) received fluticasone 200mg bid, and group B (21 males / 19 females, aged 11.5±1.8 years) clarithromycine 15 mg/kg bid, in addition to fluticasone 100 mg bid for 8 weeks. (FEV1%, C-CAT, SABA use, sputum induced % of eosinophils and neutrophils) were compared before and after treatment in each group.

## Results

In group A there is significant reduction of eosinophils percentage after treatment, and non significant increase in neutrophils percentage. There was significant improvement in FEV1% predicted. While in group B there was non significant decrease in eosinophils, and significant decrease in neutrophils. In group A there was significant negative correlation between changes in FEV1% and change in eosinophils and week positive correlation between changes in FEV1% and changes in neutrophils. In group B there was significant positive correlation between basal eosinophils and change in FEV1% and significant negative correlation between basal neutrophils and change in FEV1%.

## Conclusion

Steroids were effective in targeting eosinophilic inflammation and clarithromycine target neutrophilic inflammation. High eosinophils and neutrophils percentage in sputum are best predictors of response to steroids or clarithromycine treatment respectively.

